# 41 An Examination of Factors Predicting Inpatient Rehabilitation Stay After Burn Injury

**DOI:** 10.1093/jbcr/irad045.015

**Published:** 2023-05-15

**Authors:** Lori Turgeon, Vanessa Dellheim, Audrey O'Neil, Jeffrey Schneider, Lauren J Shepler, Alyssa Bamer, Colleen Ryan, Lewis Kazis, Karen Kowalske, oscar Suman-Vejas, Caitlin Orton, Barclay Stewart

**Affiliations:** Shriners Children's Boston, Boston, Massachusetts; Massachusetts General Hospital, saugus, Massachusetts; Richard M. Fairbanks Burn Center, Indianapolis, Indiana; Spaulding Rehabilitation Hospital/Harvard Medical School/Rehabilitation Outcomes Center at Spaulding, Boston, Massachusetts; Spaulding Rehabilitation Hospital, Harvard Medical School, Boston, Massachusetts; University of Washington, Golden, Colorado; Massachusetts General Hospital, Boston, Massachusetts; Boston University School of Public Health, Rehabilitation Outcomes Center at Spaulding, Boston, Massachusetts; UT Southwestern Medical Center, Dallas, dallas, Texas; The University of Texas Medical Branch, Galveston, Texas; University of Washington, Seattle, Washington; University of Washington, Seattle, Washington; Shriners Children's Boston, Boston, Massachusetts; Massachusetts General Hospital, saugus, Massachusetts; Richard M. Fairbanks Burn Center, Indianapolis, Indiana; Spaulding Rehabilitation Hospital/Harvard Medical School/Rehabilitation Outcomes Center at Spaulding, Boston, Massachusetts; Spaulding Rehabilitation Hospital, Harvard Medical School, Boston, Massachusetts; University of Washington, Golden, Colorado; Massachusetts General Hospital, Boston, Massachusetts; Boston University School of Public Health, Rehabilitation Outcomes Center at Spaulding, Boston, Massachusetts; UT Southwestern Medical Center, Dallas, dallas, Texas; The University of Texas Medical Branch, Galveston, Texas; University of Washington, Seattle, Washington; University of Washington, Seattle, Washington; Shriners Children's Boston, Boston, Massachusetts; Massachusetts General Hospital, saugus, Massachusetts; Richard M. Fairbanks Burn Center, Indianapolis, Indiana; Spaulding Rehabilitation Hospital/Harvard Medical School/Rehabilitation Outcomes Center at Spaulding, Boston, Massachusetts; Spaulding Rehabilitation Hospital, Harvard Medical School, Boston, Massachusetts; University of Washington, Golden, Colorado; Massachusetts General Hospital, Boston, Massachusetts; Boston University School of Public Health, Rehabilitation Outcomes Center at Spaulding, Boston, Massachusetts; UT Southwestern Medical Center, Dallas, dallas, Texas; The University of Texas Medical Branch, Galveston, Texas; University of Washington, Seattle, Washington; University of Washington, Seattle, Washington; Shriners Children's Boston, Boston, Massachusetts; Massachusetts General Hospital, saugus, Massachusetts; Richard M. Fairbanks Burn Center, Indianapolis, Indiana; Spaulding Rehabilitation Hospital/Harvard Medical School/Rehabilitation Outcomes Center at Spaulding, Boston, Massachusetts; Spaulding Rehabilitation Hospital, Harvard Medical School, Boston, Massachusetts; University of Washington, Golden, Colorado; Massachusetts General Hospital, Boston, Massachusetts; Boston University School of Public Health, Rehabilitation Outcomes Center at Spaulding, Boston, Massachusetts; UT Southwestern Medical Center, Dallas, dallas, Texas; The University of Texas Medical Branch, Galveston, Texas; University of Washington, Seattle, Washington; University of Washington, Seattle, Washington; Shriners Children's Boston, Boston, Massachusetts; Massachusetts General Hospital, saugus, Massachusetts; Richard M. Fairbanks Burn Center, Indianapolis, Indiana; Spaulding Rehabilitation Hospital/Harvard Medical School/Rehabilitation Outcomes Center at Spaulding, Boston, Massachusetts; Spaulding Rehabilitation Hospital, Harvard Medical School, Boston, Massachusetts; University of Washington, Golden, Colorado; Massachusetts General Hospital, Boston, Massachusetts; Boston University School of Public Health, Rehabilitation Outcomes Center at Spaulding, Boston, Massachusetts; UT Southwestern Medical Center, Dallas, dallas, Texas; The University of Texas Medical Branch, Galveston, Texas; University of Washington, Seattle, Washington; University of Washington, Seattle, Washington; Shriners Children's Boston, Boston, Massachusetts; Massachusetts General Hospital, saugus, Massachusetts; Richard M. Fairbanks Burn Center, Indianapolis, Indiana; Spaulding Rehabilitation Hospital/Harvard Medical School/Rehabilitation Outcomes Center at Spaulding, Boston, Massachusetts; Spaulding Rehabilitation Hospital, Harvard Medical School, Boston, Massachusetts; University of Washington, Golden, Colorado; Massachusetts General Hospital, Boston, Massachusetts; Boston University School of Public Health, Rehabilitation Outcomes Center at Spaulding, Boston, Massachusetts; UT Southwestern Medical Center, Dallas, dallas, Texas; The University of Texas Medical Branch, Galveston, Texas; University of Washington, Seattle, Washington; University of Washington, Seattle, Washington; Shriners Children's Boston, Boston, Massachusetts; Massachusetts General Hospital, saugus, Massachusetts; Richard M. Fairbanks Burn Center, Indianapolis, Indiana; Spaulding Rehabilitation Hospital/Harvard Medical School/Rehabilitation Outcomes Center at Spaulding, Boston, Massachusetts; Spaulding Rehabilitation Hospital, Harvard Medical School, Boston, Massachusetts; University of Washington, Golden, Colorado; Massachusetts General Hospital, Boston, Massachusetts; Boston University School of Public Health, Rehabilitation Outcomes Center at Spaulding, Boston, Massachusetts; UT Southwestern Medical Center, Dallas, dallas, Texas; The University of Texas Medical Branch, Galveston, Texas; University of Washington, Seattle, Washington; University of Washington, Seattle, Washington; Shriners Children's Boston, Boston, Massachusetts; Massachusetts General Hospital, saugus, Massachusetts; Richard M. Fairbanks Burn Center, Indianapolis, Indiana; Spaulding Rehabilitation Hospital/Harvard Medical School/Rehabilitation Outcomes Center at Spaulding, Boston, Massachusetts; Spaulding Rehabilitation Hospital, Harvard Medical School, Boston, Massachusetts; University of Washington, Golden, Colorado; Massachusetts General Hospital, Boston, Massachusetts; Boston University School of Public Health, Rehabilitation Outcomes Center at Spaulding, Boston, Massachusetts; UT Southwestern Medical Center, Dallas, dallas, Texas; The University of Texas Medical Branch, Galveston, Texas; University of Washington, Seattle, Washington; University of Washington, Seattle, Washington; Shriners Children's Boston, Boston, Massachusetts; Massachusetts General Hospital, saugus, Massachusetts; Richard M. Fairbanks Burn Center, Indianapolis, Indiana; Spaulding Rehabilitation Hospital/Harvard Medical School/Rehabilitation Outcomes Center at Spaulding, Boston, Massachusetts; Spaulding Rehabilitation Hospital, Harvard Medical School, Boston, Massachusetts; University of Washington, Golden, Colorado; Massachusetts General Hospital, Boston, Massachusetts; Boston University School of Public Health, Rehabilitation Outcomes Center at Spaulding, Boston, Massachusetts; UT Southwestern Medical Center, Dallas, dallas, Texas; The University of Texas Medical Branch, Galveston, Texas; University of Washington, Seattle, Washington; University of Washington, Seattle, Washington; Shriners Children's Boston, Boston, Massachusetts; Massachusetts General Hospital, saugus, Massachusetts; Richard M. Fairbanks Burn Center, Indianapolis, Indiana; Spaulding Rehabilitation Hospital/Harvard Medical School/Rehabilitation Outcomes Center at Spaulding, Boston, Massachusetts; Spaulding Rehabilitation Hospital, Harvard Medical School, Boston, Massachusetts; University of Washington, Golden, Colorado; Massachusetts General Hospital, Boston, Massachusetts; Boston University School of Public Health, Rehabilitation Outcomes Center at Spaulding, Boston, Massachusetts; UT Southwestern Medical Center, Dallas, dallas, Texas; The University of Texas Medical Branch, Galveston, Texas; University of Washington, Seattle, Washington; University of Washington, Seattle, Washington; Shriners Children's Boston, Boston, Massachusetts; Massachusetts General Hospital, saugus, Massachusetts; Richard M. Fairbanks Burn Center, Indianapolis, Indiana; Spaulding Rehabilitation Hospital/Harvard Medical School/Rehabilitation Outcomes Center at Spaulding, Boston, Massachusetts; Spaulding Rehabilitation Hospital, Harvard Medical School, Boston, Massachusetts; University of Washington, Golden, Colorado; Massachusetts General Hospital, Boston, Massachusetts; Boston University School of Public Health, Rehabilitation Outcomes Center at Spaulding, Boston, Massachusetts; UT Southwestern Medical Center, Dallas, dallas, Texas; The University of Texas Medical Branch, Galveston, Texas; University of Washington, Seattle, Washington; University of Washington, Seattle, Washington; Shriners Children's Boston, Boston, Massachusetts; Massachusetts General Hospital, saugus, Massachusetts; Richard M. Fairbanks Burn Center, Indianapolis, Indiana; Spaulding Rehabilitation Hospital/Harvard Medical School/Rehabilitation Outcomes Center at Spaulding, Boston, Massachusetts; Spaulding Rehabilitation Hospital, Harvard Medical School, Boston, Massachusetts; University of Washington, Golden, Colorado; Massachusetts General Hospital, Boston, Massachusetts; Boston University School of Public Health, Rehabilitation Outcomes Center at Spaulding, Boston, Massachusetts; UT Southwestern Medical Center, Dallas, dallas, Texas; The University of Texas Medical Branch, Galveston, Texas; University of Washington, Seattle, Washington; University of Washington, Seattle, Washington

## Abstract

**Introduction:**

Rehabilitation services improve the functional recovery of people who have experienced burn injury. Therapy services are often initiated during patients’ hospitalization and continue for months to years after discharge. However, numerous factors contribute to a patient’s overall complexity and functional impairments following discharge and need for Inpatient Rehabilitation (IR). Characteristics associated with longer IR stays have not been well explored. This study aimed to examine patient and clinical factors associated with length of IR stay. We hypothesized that burn severity, including size and location, as well as insurance payor will be associated with length of rehabilitation time.

**Methods:**

Adult participants in Burn Model System National Database (1994-2022) who were discharged to IR following their hospital stay were included. A negative binomial regression analysis examined the association between IR days and demographic and injury characteristics including age, gender, burns to critical areas (head and neck, perineum, hand), payor, etiology, circumstance of injury, and range of motion deficits. A p-value less than 0.05 was considered significant.

**Results:**

The study included 585 participants who were 72% male with mean age 46.1 (SD 16.8), and mean burn size 34.6% (20.5) total body surface area (TBSA). Average IR days were 22.8 (19.3). For every 1% increase in TBSA burned there was a 1% increase in IR days. Similarly, each additional 1 ventilator day was associated with an 1% increase in IR days. Electrical injuries were associated with 44% more IR days than flame injuries. Self-inflicted injuries were associated with 55% more IR days, while hand burns were associated with 18% decrease in IR days. Self-pay, philanthropy or other insurance coverage was associated with 37% fewer IR days. Of note, range of motion deficits were not significantly associated with IR days.

**Conclusions:**

Longer IR stays were associated with greater burn severity, etiology (electrical), and circumstance (self-inflicted), all of which are markers of overall case complexity. Shorter IR stays were associated with specific injury location (hand), though reasoning for this needs further investigation. Payor type was also associated with shorter IR stay, which highlights risk of inequity in rehabilitation service delivery.

**Applicability of Research to Practice:**

These findings can help clinicians set expectations regarding IP for patients and families, and advocate for strategies to deliver rehabilitation services more equitably.

